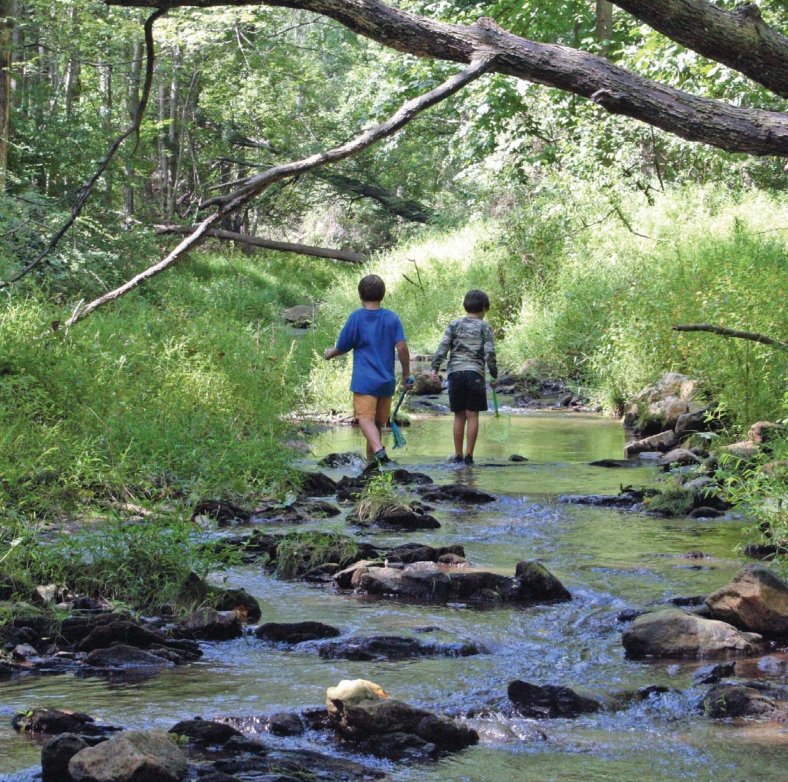# Wild Child: Guiding theYoung Back to Nature

**DOI:** 10.1289/ehp.116-a436

**Published:** 2008-10

**Authors:** Tim Lougheed

Having already tested his parents’ indulgence by adopting snakes as companions, young Ed Wilson pressed his luck even further by raising black widow spiders, which he hand-fed with live insects. By the time he took up farming ants, it must have come as something of a relief for the household.

Ed, of course, went on to become E.O. Wilson, the Harvard entomologist whose passion for creepy crawlers spawned a lifelong commitment to understanding the intricate connections between all life on Earth. Now approaching 80, he remains a leading figure in the field of systems biology, which studies the complex networks formed by the interaction of biological systems. Wilson’s childhood experience provides the archetypal account of raising a field biologist—one with hands perpetually dirty from poking into wild nooks and crannies, seeking to know what lives there. And the scientific community owes much to his early and enthusiastic attention to that world, a colorful example of the spirit embodied by so many scientists whose childhood pastimes developed into careers spent advancing our understanding of the natural world.

North America has a long tradition of nurturing this spirit, as expressed in the writings of individuals from John James Audubon to Rachel Carson. Yet the past three decades have witnessed major societal and technological changes that have transformed the way in which many young North Americans encounter nature. Where Wilson’s 1930s home might have held nothing more electronic than a crystal radio to lure him indoors, the twenty-first century equivalent can offer hundreds of television channels, increasingly vibrant computer games, kaleidoscopic Internet access, and endless social interaction to be found on glowing monitors.

Personal travel has evolved just as dramatically, so that many children spend much of their time enclosed in vehicles, often being shuttled from one indoor activity to another, perhaps without even glancing up from a handheld game or cell phone. They may well have toured airports and shopping malls on both sides of the country by the time they are teenagers, without ever having wandered among the trees left in an undeveloped lot down the street—if their neighborhood even has such property. Nor would they be encouraged to wander in such a fashion, warned of the threat posed by hostile strangers or the even more hostile legal liabilities associated with any injury. The result of this confluence of factors can be what author Richard Louv called “nature-deficit disorder” in his 2005 book *Last Child in the Woods*, a term that refers to the psychological and physical toll exacted as we become alienated from the natural environment.

These profound lifestyle and societal changes are prompting questions from researchers about what could be an equally profound change in how many North American children perceive the natural world. Of course not all children spend all their time indoors, nor is technology inherently bad; nevertheless, some observers are voicing distinctly practical concerns. If a substantial proportion of the population has little or no direct interaction with pristine natural environments as children, how will that affect their lifelong attitude toward such places? How will they come to regard the value of environmental science or policy? Above all, what kind of environmental scientists and policy managers will they become, if such careers even occur to them?

## Nature, Twenty-First Century Style?

Mark Hafner fears he may already know the answer, and it haunts him. A mammalogist with the Department of Biological Sciences and Museum of Natural Science at Louisiana State University in Baton Rouge, he regularly addresses audiences about a telling challenge he has dubbed “hantavirus hysteria.” In the early 1990s, several human fatalities caused by respiratory infection were linked with exposure to a species of hantavirus resident in native rodent populations in the U.S. Southwest. Mammalogists working with these animals were advised by the Centers for Disease Control and Prevention (CDC) and later required to outfit themselves with disposable gowns, respirators, goggles, and latex gloves to prevent infection.

Experienced researchers laughed off the gear as “moon suits”—and later epidemiologic analysis such as a report by Charles F. Fulhorst et al. in the April 2007 *Emerging Infectious Diseases* largely disproved the likelihood of becoming infected with hantavirus in the field—but Hafner insists that such recommendations have made younger newcomers to mammalogy distinctly nervous. For students who may be coming to the discipline without having spent much time in uncontrolled outdoor settings, he suggests, field work may mark the first time they have touched a wild animal. Moreover, he asserts, as the amount of gadgetry available to students has increased, their interest in the broader aspects of field work (such as collecting specimens) has decreased. As he puts it, “They’re so much more comfortable around equipment. Many of them would panic at the idea of being out of cell phone range.”

For Hafner, as for many scientists, field work often marks the highest points of his life. Eager to share the joy, he annually invites undergraduate students to join him on field trips. But each year he has been getting fewer and fewer takers, while the handful who do so remain unsure of the objective. “They just go along as passive participants, not someone who’s training to do this themselves in the future,” he says. “I just don’t know who’s going to be sampling natural populations of rodents in the future.” Hafner sees a real problem rooted in successive generations of children deprived of meaningful experiences in the natural world.

He is not alone in his assessment. In the September 2006 issue of the *Journal of Environmental Management*, Oliver Pergams and Patricia Zaradic introduced the concept of “videophilia,” which they defined as “the new human tendency to focus on sedentary activities involving electronic media.” Employing U.S. national park visitation as their bellwether, they found the steady decline of such visits since 1988 closely matched the growing prevalence and diversity of electronic entertainment options such as television, video games, and the Internet. The researchers point to census figures revealing a substantial and continuing increase in the amount of time people spend at these pastimes—an additional 327 hours per year in 2003 compared with 1987. Rising oil prices also showed a significant correlation with declining park visits.

In a study published in the 19 February 2008 issue of *Proceedings of the National Academy of Sciences*, Pergams and Zaradic expanded their data analysis to include other U.S. public lands, the issuance of hunting and fishing licenses, time spent camping or hiking, and visits to parks in Japan and Spain. The findings reinforced their earlier conclusion: even with an undiminished capacity of wild areas to handle more traffic, a smaller percentage of visitors have actually been showing up. Their analysis of 16 data sets on this kind of activity showed an average annual drop of more than 1% since the 1980s, for a total drop of 18–25% to date. “Regardless of the root cause,” they wrote, “the evidence for a pervasive and fundamental shift away from nature-based recreation seems clear.”

Commenting on their work in that same issue of the journal, Nature Conservancy chief scientist Peter Kareiva suggested this shift could well be the most serious environmental threat facing the world today. “In the end, the fate of biodiversity and ecosystems depends on political choices and individual choices,” he wrote, referring to a grasp of the vital role of natural systems in our own welfare, both physical and psychological. “If people never experience nature and have negligible understanding of the services that nature provides, it is unlikely people will choose a sustainable future.”

A parallel stream of research indicates that the foundations of those choices lie in childhood. Just as Pergams and Zaradic were homing in on videophilia, Cornell University psychologists Nancy Wells and Kristi Lekies conducted an ambitious national survey of 2,000 adults aged 18–90 to determine how childhood experiences in the natural world influenced a person’s outlook on environmental issues. “When children become truly engaged with the natural world at a young age, the experience is likely to stay with them in a powerful way—shaping their subsequent environmental path,” they reported in volume 16, issue 1 (2006) of *Children, Youth and Environments*.

## When Only the Real Thing Will Do

The research by Wells and Lekies carefully noted that the lifelong impact was more profound when the engagement with nature was spontaneous and unstructured, as characterized by the general unpredictability of pursuits such as hunting, fishing, or simply wandering around a forest. In contrast, participation in organized outdoor programs or environmental education courses did not serve as an equivalent predictor of adult attitudes toward the environment. The results even offered instances where enforced, overly structured time spent in nature with other children could later generate negative attitudes.

Pergams regularly witnesses the intricate dynamics of the personal experience with nature. In addition to being a faculty member at the University of Illinois at Chicago, he is also co-steward of a nearby nature preserve, where he takes groups of elementary school students on outings. There, he says, these young people learn that “wild” means just that—something that does not conform to the neat outline of a curriculum or the logical narrative of even the best documentary, something the children must come to grips with on their own.

“When you go out into nature, it’s a chaotic system,” Pergams says. “What engages people, especially children, is that the chaos is open-ended.” He adds that it might be tempting to re-create this response using ever more sophisticated multimedia technology. A strategically placed web camera in one of those underused national parks, for example, could usher remote wilderness locales into any place with Internet connectivity. This approach could in principle transcend the costs and logistical difficulties of bringing people to any given site, opening up the whole world to virtual exploration.

And yet, virtual is apparently not good enough, according to work published in May 2008 in the *Journal of Environmental Psychology*. Researchers tracked the heart rate of test subjects, conducting mildly stressful mental exercises in three different simulated office settings: one with a window looking out on a natural scene, another with a comparably sized high-definition television display showing a similar scene, and a third with just a blank wall.

When individuals had access to the window, their heart rate dropped. But when the television image was substituted, stress levels as indicated by pulse rates were no different than if subjects were working in a windowless room. The message, to Pergams, is that the natural world is just too chaotic for our technology to convey. “It’s almost infinite in its complexity,” he maintains. “All simulations, to whatever extent, are merely reductions of that.”

In fact, there may be even more going on than we realize whenever we are immersed in these natural settings. The “savannah hypothesis” proposed in 1980 by behavioral ecologist Gordon Orians suggests that people have an innate preference for landscape features reminiscent of the African savannahs where humans are believed to have evolved—features such as glades of wide-canopied trees, open grasslands, and scattered bodies of water. As researchers have sought to pin down this notion, their mounting evidence points to specific physical interactions. In multiple studies by geographer Roger S. Ulrich, patients recovering from surgery did so more quickly when their hospital room looked out on pleasant parkland. Another study by psychologists Frances E. Kuo and Andrea Faber Taylor, published in the September 2004 issue of the *American Journal of Public Health*, showed that the treatment of children with attention deficit/hyperactivity disorder became more effective when combined with outdoor activities. Elsewhere, studies by biological scientist Jules Pretty and colleagues have shown the benefits of exercise to be enhanced when it is performed outside.

None of this surprises Howard Frumkin, director of the CDC National Center for Environmental Health and the Agency for Toxic Substances and Disease Registry. “For the last generation or two, from the time of Rachel Carson, the field of environmental health has been very focused on protecting people from dangerous or toxic exposures,” he explains. “That’s all well and good; we need to do that. But we seem to have forgotten that environmental health can also be a positive effort rather than a negative effort, an effort to promote good environments rather than control bad environments. There’s an awakening in the last few years to the fact that there is this other side to the coin, and it’s a very powerful concept. One wonders why we didn’t get this earlier.”

## In Search of Wild Children

So where will the next generation of environmental health scientists come from? The CDC began answering that question this year with its new Collegiate Leaders in Environmental Health internship program. The program exposes undergraduates to a broad overview of environmental and public health issues at the federal level and lets them participate in environmental health projects, interact with federal officials and scientists, and visit important environmental health sites in Atlanta. Frumkin recalls that the plan started with 10 of these posts, but after receiving upward of 200 outstanding applicants the number was raised to 12. If that outcome bodes well for the institutional needs of the CDC, it also inspires Frumkin to see even more that can be done. “We very much need to be creating the next generation of environmental health professionals, but more broadly than that, citizens who care about the environment,” he says.

That call strikes a chord that is echoed in a number of different venues. Some build on the momentum of academic work, like the Red Rock Institute, a Pennsylvania-based nonprofit corporation founded by Pergams and Zaradic to pursue the implications of videophilia and its possible solutions. Meanwhile, grassroots concerns have spawned the Safe Routes to Schools movement in California. Citing CDC figures showing that 50% of children walked or bicycled to school in 1969 whereas only 15% do so today, this group promotes the restoration of outdoor activity. A similar sentiment has inspired Representative John Sarbanes (D–MD) to champion No Child Left Inside, an amendment to the 1965 Elementary and Secondary Education Act that would assign environmental awareness a much more prominent place in schools.

Of such programs, Louv says, “It’s terrific to see such an array of ideas about how to connect children to nature. We may not agree on all of them, but what’s most encouraging is that we’re having this discussion, nationally and internationally, and that this issue has the peculiar property of transcending political and religious differences.”

Louv stresses that the process of reconnecting children with nature should not be premised on trying to distance children from the beloved technology that would appear to be alienating them from nature in the first place (a traditional recipe for failure, as parents discover in criticizing their children’s choice in music). Electronics are a factor, he says, but he regards the real sources of this alienation as the disappearance of natural play, societal fear of strangers and legal liability associated with injury, and the excessive structure imposed on children’s development.

Even the simple act of loosing children for a few minutes during the school day is regarded by some as an indulgence. As of 2001, the National Association of Early Childhood Educational Specialists in State Departments of Education was sounding the alarm on the decline of recess in schools. In some 40% of the 16,000 U.S. school districts this traditional outdoor break had been eliminated or was being considered for elimination. Much of the rationale for these changes had been summed up a few years earlier by Benjamin O. Canada, the superintendent of Atlanta schools, who was quoted in the 7 April 1998 *New York Times* as saying, “We are intent on improving academic performance. You don’t do that by having kids hanging on the monkey bars.” More recently, other schools have banned games such as tag and soccer, citing concerns that such forms of play lead to aggression between children and lawsuits if kids get hurt.

But for many children, recess can be one of only a few chances in a highly structured day to connect with the natural world. And Louv insists children have a right to that connection. “It is a fundamental part of their humanity, and ours,” he says. “If we don't pass this on to our children and grandchildren, who will? How many generations will go by before our cultural memory fades?”

Wilson puts these very questions to his own colleagues in his 2006 book *The Creation*. There he reminds them that as important and alluring as they may find the frontiers of laboratory sciences like molecular biology and neurochemistry, the larger living world around us remains the richest and most relevant ground for discovery.

“From the freedom to explore comes the joy of learning,” he wrote. “From knowledge acquired by personal initiative arises the desire for more knowledge. And from mastery of the novel and beautiful world awaiting every child comes self-confidence. The growth of a naturalist is like the growth of a musician or athlete: excellence for the talented, lifelong enjoyment for the rest, benefit for humanity.”

## Figures and Tables

**Figure:**